# Covalent-Organic Framework with Unconventional D-D Structure for Efficient Photocatalytic Uranium Extraction

**DOI:** 10.3390/molecules31132263

**Published:** 2026-06-26

**Authors:** Dongyang Xu, Xin Du, Bingyue Zhou, Lixi Chen, Mengyao Li, Qiang Wu, Jun Liu, Songbai Tang, Guowen Peng

**Affiliations:** 1 Key Laboratory of Nuclear Facility Decommissioning and Ecological Restoration of the Ministry of Ecology and Environment, School of Resources Environment and Safety Engineering, Hunan Provincial Engineering Research Center for Safety Control and Recycling of Radioactive Heavy Metal Pollutants, University of South China, 28 Changsheng West Road, Hengyang 421001, China; xdy17395861820@163.com; 2 State Key Laboratory of Radiation Medicine and Protection, School of Radiation Medicine and Protection, Collaborative Innovation Center of Radiological Medicine of Jiangsu Higher Education Institutions, Biomedical Basic Research Center (BBRC) of Jiangsu, Soochow University, Suzhou 215123, China; 20254220023@stu.suda.edu.cn (X.D.); 20254020010@stu.suda.edu.cn (B.Z.); lxchen@suda.edu.cn (L.C.); 2530509112@stu.suda.edu.cn (M.L.); 3 Key Laboratory of Advanced Nuclear Energy Design and Safety, Ministry of Education, University of South China, 28 Changsheng West Road, Hengyang 421001, China

**Keywords:** radioactive wastewater, donor-donor covalent organic frameworks (D-D COFs), photocatalysis, uranium extraction

## Abstract

Photocatalytic extraction of uranium from radioactive wastewater is crucial for environmental safety and sustainable nuclear energy development. It is widely recognized that photocatalysts with donor-acceptor (D-A) or D-π-A structures exhibit enhanced charge separation efficiency, thereby showing excellent photocatalytic performance. Herein, we presented a counterintuitive design of a donor-donor covalent-organic framework (D-D COF) for efficient photocatalytic uranium extraction. A twisted D-D COF (COF-BCTB-Py) was synthesized via solvothermal condensation using bicarbazole and pyrene as dual electron-donor units. The COF featured a well-defined AA-stacked porous structure, high specific surface area (963 m^2^·g^−1^), suitable band gap (2.44 eV), and exceptional chemical, thermal, and radiation stability. Impressively, in the presence of 5% methanol, it delivered an ultrahigh uranium uptake capacity of 4278 mg·g^−1^ with fast kinetics and >97% removal efficiency in complex water matrices, challenging the traditional stereotype of low-activity D-D COFs. Mechanistic studies revealed that soluble U(VI) was converted into crystalline (UO_2_)O_2_·2H_2_O via in situ generated hydrogen peroxide rather than being reduced to U(IV). This work provides an unconventional design strategy to design efficient photocatalysts for uranium recovery from nuclear wastewater.

## 1. Introduction

Nuclear energy has emerged as one of the cornerstone strategies for achieving global carbon neutrality, constituting ~one-third of global low-carbon electricity generation [1].

An ambitious goal has been projected by the World Nuclear Association to expand the global nuclear capacity from 391 gigawatts (GW) to 686 GW by 2040 [2]. Nevertheless, behind the rapid growth lies the generation of substantial volumes of uranium-laden effluents, which pose dual toxicity derived from its radioactivity and chemical hazard [3]. Once released into the environment, uranium typically exists in the soluble uranyl form (UO_2_^2+^) [4], which can be transported via rivers and groundwater, posing severe risks to human health and the ecological environment [5]. According to the regulations stipulated by the United States Environmental Protection Agency (EPA), uranium concentrations in potable water must not exceed 30 ppb, otherwise it may damage kidneys, bones, and DNA [6]. Therefore, the recovery and extraction of uranium from radioactive wastewater are deemed essential not only for environmental sustainability and human health, but also for the conservation of nuclear energy resources.

To date, several approaches have been developed to extract and recover uranium from wastewater, including chemical precipitation [7], solvent extraction [8], adsorption [9,10], ion exchange [11], electrocatalysis [12,13,14,15,16], photocatalysis [17,18,19,20], and so on. Chemical precipitation enables rapid uranium immobilization but suffers from poor selectivity, narrow pH tolerance, and secondary pollution from chemical additives. Conventional solvent extraction and adsorption rely on organophosphorus reagents, requiring multi-stage steps that cause high reagent and solvent loss. Achieving high selectivity also remains difficult for adsorption and ion exchange, especially in complex matrices. Electrochemical methods offer simplicity and fast kinetics, yet face scalability issues due to high energy demand, slow mass transfer, and electrode deactivation. Photocatalysis offers a viable route for uranium recovery, achieved by converting soluble U(VI) to insoluble states such as UO_2_ or (UO_2_)O_2_·2H_2_O. This protocol features low additive usage, operational simplicity, and alignment with green chemistry principles. Various photocatalysis systems have been developed using inorganic or organic photocatalysts, such as metal oxides [21,22,23], sulfides [24,25,26,27], g-C_3_N_4_ [28,29,30], metal-organic frameworks (MOFs) [31,32,33,34], and covalent–organic frameworks (COFs) [35,36,37,38]. Compared with traditional catalysts, COFs exhibit exceptional resistance to radiation and harsh acidity (typical of uranium-containing wastewater at pH < 4) owing to the structural robustness conferred by their covalent linkages and π-conjugation. The photophysical and electronic properties can be precisely tuned by modulating the linkage chemistry [18], incorporating functional moieties [35,38], doping with heteroatoms [39], and implementing structural or defect engineering [27]. Since the first COF reported in 2005 by Yaghi and coworkers [40], these porous polymers have become versatile platforms for photocatalysis [41,42,43,44,45], while applications in photocatalytic uranium uptake only emerged circa 2020, leaving ample scope for further optimization [17].

Generally, COFs with a donor-acceptor (D-A) [20,46] or donor-π-acceptor (D-π-A) [47,48,49,50,51,52] structure show admirable photocatalytic uranium separation performance because the electron structure of these COFs facilitates conjugation extension, electron transport, and enhanced charge separation efficiency. In applications of uranium extraction, Pei et al. [20] pioneered 5,10,15,20-tetra(4-aminophenyl) porphyrin (TAPP) as the electron donor and 2,5-dihydroxy-1,4-benzoquinone (DHBQ) as the electron acceptor, achieving a remarkable 98.3% uranium removal rate under acidic conditions (pH = 4.5). Meanwhile, Huang et al. [46] proposed a site engineering strategy using 1,3,5-triformylphloroglucinol (Tp) as the electron acceptor and 2,6-diaminoanthraquinone (DAAQ) as the electron donor. The resulting β-ketoenamine linkage improved π-electron delocalization and chemical stability, achieving an exceptional 99.2% U(VI) removal ratio without sacrificial agents. For D-π-A-structured COFs, the insertion of a π-bridge between donor and acceptor units offers a versatile strategy to modulate framework coplanarity and exciton binding energy. Wang et al. [51] demonstrated that a naphthalene π-bridge achieves an optimal dihedral angle of only 1.90°, thereby establishing a D-π-A structure featuring a planar delocalized electron transport pathway that reduces the exciton binding energy to 57.42 meV and significantly boosts U(VI) photoreduction efficiency. Similarly, Liu et al. [52] constructed carboxyl-functionalized quinoline-linked D-π-A COFs via the Doebner reaction, where the extended π-conjugation and additional charge transfer channels lowered the band gap to 1.60 eV, enabling sacrificial-agent-free uranium extraction with over 97% efficiency. Research on COF-based photocatalytic uranyl removal has predominantly focused on D-A and D-π-A architectures, while other structures such as donor-π-donor (D-D) configurations remain largely unexplored. Extending the structural diversity of COFs represents a promising strategy to uncover novel photophysical properties and unique charge delocalization dynamics for efficient photocatalytic uranium removal.

Herein, we report a special conjugation-hindered D-D COF consisting of a twisted bicarbazole-based building block (BCTB) and a highly conjugated 1,3,6,8-tetraphenylpyrene counterpart linker (Py). Through the structural modulation of the distorted BCTB, this material exhibits an appropriate band gap (2.44 eV) and is capable of photocatalytic removal of uranium, leading to a superior removal efficiency in natural water matrices (97%) and super high capacity (4278 mg·g^−1^). Notably, this COF exhibits exceptional tolerance to ionizing radiation (up to 50 kGy) and a wide pH range (4–7), demonstrating the potential for treatment in actual radioactive wastewater.

## 2. Results

**Figure 1:**
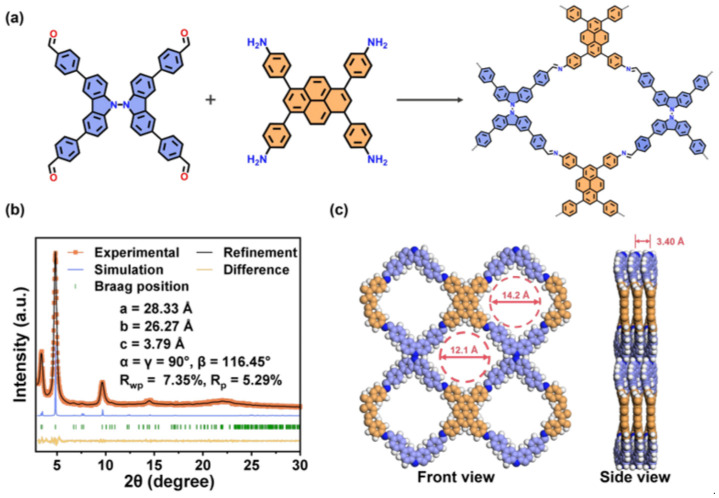
(**a**) Schematic illustration of the synthesis of COF-BCTB-Py via solvothermal reaction at 120 °C for 3 days. (**b**) Experimental and theoretical powder X-ray diffraction (PXRD) patterns. (**c**) Front view and side view of COF-BCTB-Py, the dashed circles schematically illustrate the diameters of cylindrical pores at the corresponding structural segments.

Bicarbazole is widely recognized as a robust electron-donating moiety that optimizes the electronic conductivity of COF skeletons, while pyrene possesses an excellent electron-donating capacity to modulate the electronic distribution of conjugated frameworks. Herein, these two electron-rich linkers were intentionally selected to fabricate a rare D-D-type COF, realizing an unconventional structural design for photocatalytic materials. As depicted in Figure 1, COF-BCTB-Py was synthesized and purified following previously documented protocols [30]. The structural model of the target COF was constructed via Materials Studio 2021 software. Subsequent Pawley refinement revealed that the simulated PXRD pattern derived from the AA-stacking configuration exhibited excellent consistency with experimental diffraction results.

The finalized unit cell parameters of the AA-stacked COF-BCTB-Py were determined as follows (Figure 1b): a = 28.33 Å, b = 26.27 Å, c = 3.79 Å, α = γ = 90°, β = 116.45°, belonging to the P1 space group. The refinement reliability factors were calculated to be Rwp = 7.35% and Rp = 5.29%, manifesting a high coincidence between the optimized structural model and the actual crystal structure of COF-BCTB-Py. Distinct characteristic diffraction peaks were observed at (2 theta) values of 3.23°, 3.57°, 4.82°, 9.69°, 14.55° and 24.88°, which can be unambiguously assigned to the (010), (100), (110), (220), (330) and (101) crystal planes of the two-dimensional AA-stacking structure, respectively. Further structural simulation demonstrated that the interlayer distance of COF-BCTB-Py was around 3.40 Å (Figure 1c), indicative of strong π-π interactions between layers, and its major pore size was distributed in the range of 1.2–1.4 nm.

**Figure 2:**
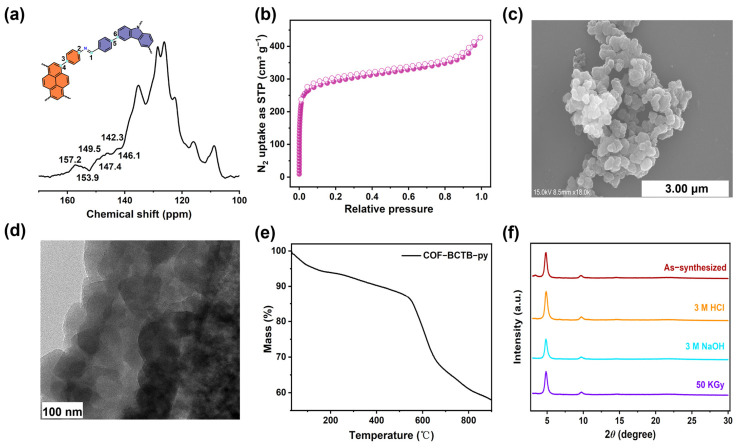
(**a**) SS-^13^C-NMR of COF-BCTB-Py, the Arabic numerals marked on the chemical structure in the figure represent the serial numbers of the adjacent carbon atoms. (**b**) Nitrogen adsorption-desorption isotherms. Inset, pore size distribution of COF-BCTB-Py. (**c**) SEM images of COF-BCTB-Py. (**d**) TEM images of COF-BCTB-Py. (**e**) Thermogravimetric analysis curves of COF-BCTB-Py under nitrogen atmosphere. (**f**) PXRD spectra of COF-BCTB-Py after 3 M HCl, 3 M NaOH immersion or 50 kGy ionizing irradiation treatments.

Solid-state ^13^C nuclear magnetic resonance (SS-^13^C-NMR) spectra of COF-BCTB-Py were recorded and are presented in Figure 2a. Benefiting from the high structural symmetry of COF-BCTB-Py, the carbon atoms within the framework possess 20 distinct chemical environments. Electron-withdrawing neighboring groups can reduce the electron cloud density of adjacent carbon sites, leading to their resonance signals shifting toward higher chemical shifts. Accordingly, the characteristic signals located at 157.2 ppm and 153.9 ppm are assigned to carbon atoms adjacent to nitrogen atoms in imine linkages (C1, C2). The peaks at 149.5, 147.4, 146.1 and 142.3 ppm with relatively higher chemical shifts correspond to the α-carbon atoms on benzene rings linked to large, conjugated moieties (C3–C6). The remaining resonance signals ranging from 100 ppm to 140 ppm are attributed to other carbon sites distributed on benzene rings and conjugated skeletons of the COF. The spectral results verify the formation of imine bonds, as well as the chemical environment evolution of original ligands, further confirming the successful fabrication of target COF-BCTB-Py. Moreover, Fourier Transform Infrared Spectroscopy (FT-IR) comparative analysis between raw monomers and final products displays a prominent new absorption band at 1631.8 cm^−1^ originating from imine stretching vibration, which further corroborates the successful construction of COF-BCTB-Py (Figure S1).

Nitrogen adsorption-desorption measurements were performed at 77 K in a liquid nitrogen bath to evaluate the specific surface area and pore structure of COF-BCTB-Py. As observed in Figure 2c, the physisorption isotherm of COF-BCTB-Py shows incomplete pore filling at low relative pressure. The sample maintains high adsorption uptake at high relative pressure, and capillary condensation gives rise to evident hysteresis, a typical feature of Type IV isotherms [53]. According to the BET calculation, the synthesized COF possesses a high specific surface area of 963 m^2^·g^−1^ and a pore volume of 0.66 cm^3^·g^−1^. Moreover, the pore size distribution was calculated using the NLDFT method. Calculations were performed with a standard cylindrical pore model for zeolites and silicon-based materials, based on N2 adsorption data collected at 77 K. The result revealed that the pore diameter of COF-BCTB-Py is centered at approximately 12 Å, which is highly consistent with the aforementioned structural simulation and thereby validates the rationality of the crystallographic model. Field-emission scanning electron microscopy (FE-SEM) and transmission electron microscopy (TEM) were employed to characterize the nanoscale morphology of COF-BCTB-Py. Prior to characterization, the sample was exfoliated in ethanol for 1 h at room temperature. The SEM images indicate that COF-BCTB-Py presents irregular bulk aggregates composed of spherical nanoparticles with diameters ranging from 400 to 450 nm (Figure 2c). Such morphological features originate from the low intrinsic density and easy grain agglomeration, which are commonly observed in two-dimensional COF materials. Furthermore, the TEM images (Figure 2d) confirm that these spherical aggregates are assembled from twisted stacked nanosheets, exhibiting an obvious clustered structure.

Thermogravimetric analysis (TGA) was performed to evaluate the thermal stability of COF-BCTB-Py. As depicted in Figure 2e, the thermogravimetric curve presents two prominent mass-loss stages near 150 °C and 560 °C. The slight weight loss (less than 5%) below 150 °C is attributed to the evaporation of adsorbed water and residual solvent molecules trapped within the porous framework. The secondary mass loss (5–15%) occurring between 150 °C and 560 °C originates from the detachment of trace oligomers and terminal capping groups. A drastic mass decline (over 30%) is observed above 570 °C, which corresponds to the cleavage of imine linkages and the structural collapse of the COF skeleton. These results confirm the excellent thermal stability of COF-BCTB-Py. For the practical application of photocatalytic uranium extraction in complex aqueous environments, chemical durability under extreme harsh conditions is an essential prerequisite. The acid-base stability of COF-BCTB-Py was investigated by soaking samples in 3 M strong acidic and alkaline media (pH < 0 and pH > 14) for 24 h. The intact characteristic diffraction peaks in the treated PXRD patterns (Figure 2f) demonstrate its superior chemical tolerance toward extreme pH conditions. Moreover, the irradiation resistance was further evaluated under an ionizing radiation dose of 50 kGy. The material retains its crystalline structure and photocatalytic performance after irradiation because of the rich aromatic rings in COF-BCTB-Py (Figure 2f and Figure S2), revealing outstanding radiation stability for harsh radioactive aqueous environments. The excellent thermal, chemical, and radiation stability of COF-BCTB-Py guarantees its application for photocatalytic uranium uptake.

**Figure 3:**
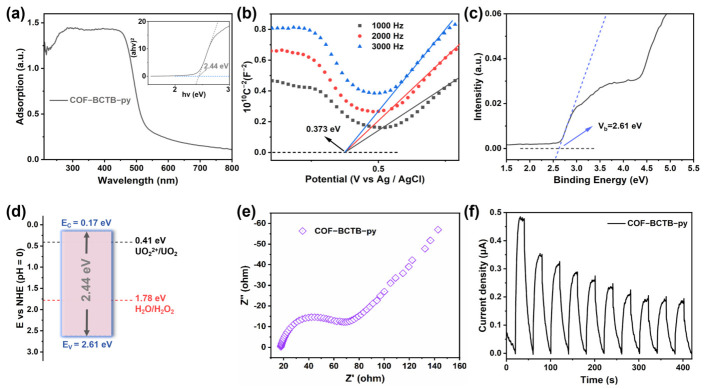
(**a**) UV-Vis diffuse reflectance spectrum of COF-BCTB-Py. Insert, the optical band gap was calculated via the Tauc plot derived from the absorption spectrum. (**b**) Mott-Schottky plot of COF-BCTB-Py for the determination of flat-band potential (E_fb_). The flat-band potential was derived from the x-intercept of the linear region of the curve. (**c**) Ultraviolet photoelectron spectroscopy of COF-BCTB-Py for determination of the valence band (V_b_). The V_b_ edge is obtained by linear extrapolation of the onset of the photoemission cutoff. (**d**) Band gap distribution diagram of COF-BCTB-Py. (**e**) Nyquist plots of COF-BCTB-Py. (**f**) Transient photocurrent curves of COF-BCTB-Py.

A series of photoelectrochemical measurements, including UV-visible diffuse reflectance spectroscopy (UV-vis-DRS), Mott-Schottky analysis, ultraviolet photoelectron spectroscopy (UPS), electrochemical impedance spectroscopy (EIS), and transient photocurrent tests, were conducted to systematically investigate the optical, electrochemical, and semiconducting properties of COF-BCTB-Py. As illustrated in Figure 3a, COF-BCTB-Py exhibits a broad optical absorption range from 200 to 560 nm, enabling efficient utilization of visible-light photons. The optical band gap was calculated to be 2.44 eV via the Tauc plot method (inset of Figure 3a). The moderate band gap of 2.44 eV enables COF-BCTB-Py to achieve balanced visible-light absorption and suitable redox potential. Such an optimal band structure not only guarantees effective photogenerated charge separation, but also thermodynamically supports the in situ generation of hydrogen peroxide, thereby facilitating the formation of (UO_2_)O_2_·2H_2_O and achieving high-efficiency photocatalytic uranium extraction. The conduction band (C_B_) position was determined based on the flat band potential (E_fb_) acquired from Mott-Schottky measurements. The positive slope of the Mott-Schottky curve in Figure 3b confirms the n-type semiconductor characteristic of COF-BCTB-Py. UPS was utilized to elucidate the surface energy level structure (Figure 3c). The Fermi level was determined by the tangent extrapolation of the UPS straight-line portion, and the corresponding valence band (E_V_) of COF-BCTB-Py was calculated to be 2.61 eV. Combined with the aforementioned band gap value, the conduction band (E_C_) was further deduced to be 0.17 eV. As summarized in Figure 3d, the energy band alignment of COF-BCTB-Py was compared with the redox potentials of the uranium-containing system. The band structure not only satisfies the thermodynamic requirement for the reduction of UO_2_^2+^ to UO_2_ (0.41 eV), but also matches the potential for water oxidation to hydrogen peroxide (1.78 eV). However, benefiting from a high valence band position, COF-BCTB-Py exhibits robust oxidation potential and enables ambient H_2_O_2_ production via water oxidation. Hence, even if UO_2_ is photoproduced over COF-BCTB-Py, the resulting uranium species can be readily re-oxidized by in situ formed H_2_O_2_ to yield (UO_2_)O_2_·2H_2_O. Consequently, COF-BCTB-Py possesses sufficient thermodynamic potential and qualifies as a promising photocatalyst for uranium extraction.

Subsequently, EIS measurements were employed to evaluate the interfacial charge transport behavior. The Nyquist plot (Figure 3e) displays a small semicircle with a diameter of approximately 40 Ω in the low-frequency region, which represents the charge transfer resistance of COF-BCTB-Py. This relatively low resistance is superior to that of most previously reported COF materials. Benefiting from the dual electron-donor units anchored on the COF skeleton, the synergistic electronic effect accelerates interfacial charge migration, thereby optimizing charge transport capability and providing a favorable foundation for enhanced photocatalytic performance. Photocurrent density serves as a critical evaluation indicator to assess the photocatalytic uranium-reduction performance of functional materials. Under visible-light irradiation with an external bias of 0.068 V vs. RHE, COF-BCTB-Py delivers a transient photocurrent density of 0.3 μA·cm^−2^ (Figure 3f), demonstrating its favorable photogenerated charge production capability. Consequently, COF-BCTB-Py possesses sufficient thermodynamic potential and qualifies as a promising photocatalyst for uranium extraction.

## 3. Discussion

**Figure 4:**
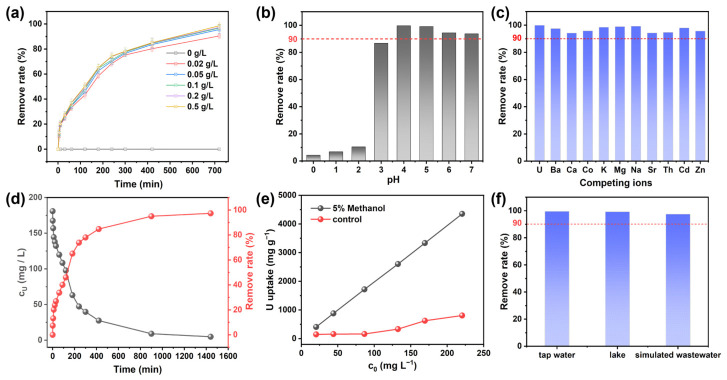
(**a**) Effect of catalyst dosage on photocatalytic uranium extraction performance (t = 720 min). (**b**) Effect of pH (0–7) value on photocatalytic uranium extraction performance. (**c**) Effect of different interfering ions on the photocatalytic performance of COF-BCTB-Py (pH = 4, C_U_ = 0.01 mmol·L^−1^, interfering ions: 1 mmol·L^−1^, 5% methanol). (**d**) Photocatalytic uranium extraction kinetic curves of COF-BCTB-Py (pH = 4, C_0_ = 180 mg·L^−1^, 5% methanol). (**e**) Photocatalytic uranium extraction isotherms of COF-BCTB-Py in the presence and absence of 5% methanol. (**f**) Photocatalytic uranium extraction performance of COF-BCTB-Py in natural water (50 mg·L^−1^ U added) and simulated uranium-containing wastewater.

The above results indicate that the as-prepared COF-BCTB-Py also possesses a suitable band gap that may also enable efficient photocatalysis. The photocatalytic uranium extraction performance of COF-BCTB-Py was systematically investigated in uranium-containing wastewater. Prior to batch photocatalytic experiments, critical experimental parameters were optimized, including the selection of hole scavengers, catalyst dosage, and solution pH. As depicted in Figure 4a, the self-degradation of uranyl ions was negligible in the blank group without catalysts. At 720 min, the removal efficiency of UO_2_^2+^ was significantly improved to 89% at 0.02 g·L^−1^ (ratio of catalyst mass over solution volume), and then gradually improved to 99.2% at 0.1 g·L^−1^. Such enhancement is attributed to the increased number of accessible active sites, which facilitates the photocatalytic reaction. Nevertheless, a further increase in dosage (0.1–0.5 g·L^−1^) failed to continuously promote uranium removal performance. Considering the comprehensive balance between catalytic efficiency and economic cost, 0.1 g·L^−1^ was ultimately selected as the optimal catalyst dosage for subsequent experiments.

The pH value of solutions would greatly influence the uranium speciation (e.g., UO_2_^2+^ at acidic conditions and UO_2_^2+^(OH)_x_^−^ at basic conditions), thereby affecting the uranium uptake performance. The effect of solution pH on uranium extraction performance was further explored (Figure 4b). The uranium removal rate sharply decreased to 10.34% under strongly acidic conditions (pH < 4). Excessive hydrogen ions can compete with uranyl ions for limited active sites on the COF surface. Additionally, the imine linkage of COF-BCTB-Py is susceptible to protonation in highly acidic environments, which hinders photoelectron migration during the photocatalytic reaction. Notably, the uranium removal efficiency remained consistently above 90% within the pH range of 4–7, manifesting the superior environmental adaptability and structural stability of COF-BCTB-Py under weakly acidic to neutral conditions. Given the weakly acidic nature of authentic uranium-containing wastewater and the significant pH dependence of the extraction performance, pH = 4 was selected as the optimal experimental condition for COF-BCTB-Py.

Under practical conditions (e.g., nuclear waste liquids or polluted environmental water), various metal ions coexist with UO_2_^2+^ and compete for binding, which may reduce uranium uptake performance. Anti-interference experiments were carried out in the presence of ten common competing metal cations at 100-fold excess concentrations relative to uranyl ions (Figure 4c). The uranium extraction rate was still maintained over 95%, confirming the prominent selectivity of COF-BCTB-Py toward uranyl ions. As shown in Figure 4d, the photocatalytic uranium removal kinetics (0.1 g L^−1^ dosage with 5% methanol) indicates that COF-BCTB-Py could remove more than 85% of uranyl ions within 7 h in the high-concentration uranium wastewater (initial U concentration = 181 mg·L^−1^). After 12 h of irradiation, the residual uranium content in the solution was reduced to less than 5%, and the maximum uranium removal capacity reached 1654.2 mg·g^−1^.

Furthermore, under fixed external conditions (pH = 4, catalyst dosage = 0.05 g·L^−1^), the uranium extraction performance of COF-BCTB-Py was evaluated for solutions with varied initial uranium concentrations. As displayed in Figure 4e, the isothermal line (0.05 g L^−1^ dosage with 5% methanol) of photocatalytic uranium removal demonstrates that the maximum uranium extraction capacity of COF-BCTB-Py increases linearly with the initial uranium concentration in the 0–220.5 ppm range, accompanied by an exceptional uptake capacity of 4278 mg·g^−1^. This remarkable value surpasses the uranium adsorption capacity of most previously reported functional materials. By contrast, the control group without methanol hole scavengers exhibited a drastic decline in both extraction efficiency and adsorption capacity. This comparison confirms that sacrificial agents are indispensable; without methanol, photogenerated charges cannot be effectively separated, thereby restricting the photocatalytic uranium extraction process even under visible-light irradiation. To further evaluate practical application feasibility, natural water matrices (including groundwater and lake water spiked with 50 mg·L^−1^ uranium) and simulated nuclear wastewater were tested under identical experimental conditions (Figure 4f). Encouragingly, the uranium extraction efficiency remained higher than 97% in these complex water systems. Such results demonstrate the outstanding practical applicability of COF-BCTB-Py, which holds great promise for uranium separation from high-concentration uranium-containing radioactive wastewater.

**Figure 5:**
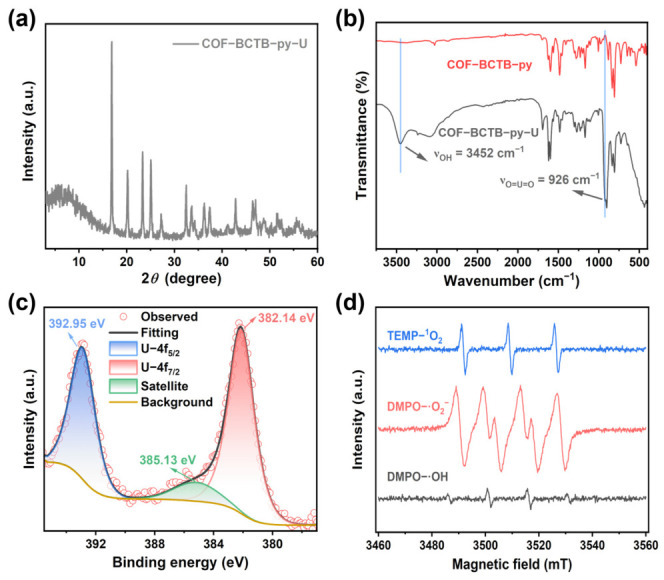
(**a**) PXRD patterns of COF-BCTB-Py after photocatalytic uranium extraction. (**b**) FT-IR spectra of COF-BCTB-Py before and after photocatalytic reaction. (**c**) High-resolution U 4f XPS spectra of COF-BCTB-Py after photocatalysis. (**d**) EPR signals of ^1^O_2_, ·O_2_^−^, and ·OH under light conditions (5 min).

PXRD, FT-IR, and XPS measurements were conducted to identify the uranium-containing precipitates and reveal the photocatalytic uranium extraction mechanism of COF-BCTB-Py under ambient conditions. As displayed in Figure 5a, the PXRD pattern of the post-reaction precipitate matches well with the standard diffraction peaks of (UO_2_)O_2_·2H_2_O. In comparison with pristine COF-BCTB-Py, the spent sample exhibits two newly generated infrared absorption bands at 926 cm^−1^ and 3452 cm^−1^, which are attributed to the ν_O=U=O_ and ν_OH_ stretching vibrations of (UO_2_)O_2_·2H_2_O, respectively (Figure 5b). Moreover, the majority of skeleton-related infrared peaks remain almost unchanged. In particular, the imine stretching vibration ν_C=N_ at 1626 cm^−1^ is clearly retained, demonstrating the excellent structural and compositional stability of COF-BCTB-Py throughout the photocatalytic process. Additionally, XPS spectra were applied to analyze the chemical state of uranium precipitates (Figure 5c). The characteristic peaks at 392.95 eV and 382.14 eV are assigned to U 4f_5/2_ and U 4f_7/2_ of U(VI), while the weak signal at 385.08 eV corresponds to the satellite peak of hexavalent uranium. Collectively, these comprehensive characterizations confirm that (UO_2_)O_2_·2H_2_O is the dominant solid uranium product formed during air-mediated photocatalytic uranium extraction [54].

Notably, neither UO_2_ nor tetravalent uranium (U(IV)) was detected in the PXRD and XPS results. Interestingly, only the characteristic diffraction peaks of (UO_2_)O_2_·2H_2_O were detected for a small amount of sediment collected from the photocatalytic uranium extraction system without methanol (Figure S3). This phenomenon can be attributed to the relatively high valence band position of COF-BCTB-Py, which renders the photocatalyst thermodynamically unfavorable for uranium reduction. Accordingly, it is speculated that in situ-generated hydrogen peroxide plays a critical role in uranium immobilization and (UO_2_)O_2_·2H_2_O formation. Electron paramagnetic resonance (EPR) spectroscopy was further performed to identify reactive oxygen species (ROS) and elucidate the radical-mediated reaction pathway (Figure 5d). The EPR spectra demonstrate that COF-BCTB-Py produces prominent ·O_2_^−^ radicals with distinct DMPO-^1^O_2_ adduct peaks under visible-light irradiation, whereas a negligible signal is detected in the dark, verifying the excellent photoinduced activity of COF-BCTB-Py. Meanwhile, 2,2,6,6-tetramethyl-4-piperidone (TMPD) was utilized as a spin trap for singlet oxygen ^1^O_2_. The detectable ^1^O_2_ signal confirms the occurrence of energy transfer originating from the extensive π-conjugated framework of COF-BCTB-Py. Moreover, photogenerated charge carriers were also monitored via EPR measurements. A distinct EPR signal centered at g = 2.03 emerges under visible-light excitation, confirming the generation of conduction-band electrons (Figure S4). In addition, recognizable DMPO−·OH adduct signals are observed, demonstrating that water or hydroxyl groups can be oxidized to produce strong oxidative hydroxyl radicals, which further facilitate endogenous hydrogen peroxide generation. Combined with the sharply declined uranium extraction efficiency without methanol and referring to the relevant mechanistic literature, the photocatalytic uranium extraction pathway in this work is proposed. Uranyl ions chemically react with in situ-generated hydrogen peroxide to form crystalline (UO_2_)O_2_·2H_2_O, following the equation:

UO_2_^2+^ + 2H_2_O + H_2_O_2_ → (UO_2_)O_2_·2H_2_O + 2H^+^ (1)

## 4. Materials and Methods

### 4.1. Materials

As illustrated in Figure 1a, 29.95 mg of 4,4′,4″,4‴-([9,9′-dicyclohexyl]-3,3′,6,6′-tetrayl) tetrabenzaldehyde (BCTB-CHO, 0.05 mmol) and 28.33 mg of 1,3,6,8-tetrakis(4-aminophenyl)pyrene (Py-NH_2_, 0.05 mmol) were weighed into a 10 mL pressure-resistant glass vial. The mixture was degassed by three freeze-pump-thaw cycles under liquid nitrogen (77 K), then sealed under vacuum. The sealed vial was heated in a forced-air oven: the temperature was ramped over 2 h to 120 °C and held for 3 days. The crude product was washed and centrifuged 3–5 times sequentially with tetrahydrofuran, acetone, and methanol, followed by drying at 80 °C in a vacuum oven for 12 h. The final product, denoted COF-BCTB-Py, was collected as a light-yellow powder with a yield of 85% (Figure S5).

### 4.2. Methods

#### 4.2.1. Model Modeling and Structural Refinement

Crystal structure modeling of COF-BCTB-Py, including construction of the AA-stacking theoretical model, simulation of powder X-ray diffraction (PXRD) patterns, and Pawley refinement, was performed using Materials Studio 20.1 (BIOVIA, 5005 Wateridge Vista Drive, San Diego, CA 92121, USA). During structural modeling, geometry optimization and energy minimization were conducted to refine the COF and obtain the relaxed equilibrium structure. For Pawley refinement, experimental PXRD data were imported and refined using the Reflex module in Materials Studio 20.1. Energy minimization was implemented with the universal force field (UFF) and the conjugate gradient algorithm. The convergence criteria were set to 0.001 kcal·mol^−1^ for energy and 0.5 kcal·mol^−1^·Å^−1^ for force, respectively.

#### 4.2.2. Characterization of Material Basic Structure, Components and Morphology

The basic structure and physicochemical properties of COF-BCTB-Py were characterized using a series of standard techniques, including PXRD, FT-IR, BET, SEM, HRTEM, AFM, and XPS. All measurements were performed following the instrument specifications and operating procedures detailed in the Supplementary Information (SI).

#### 4.2.3. Optoelectronic Physical Property Testing

In this work, EPR, EIS, Mott-Schottky plots, transient photocurrent density, and other related photoelectrochemical measurements of COF-BCTB-Py were collected using the instruments and standard testing procedures detailed in the Supplementary Information (SI).

#### 4.2.4. Uranium Removal Experiment by Photocatalysis

Photocatalytic removal of UO_2_^2+^ was investigated at different pH values to evaluate the pH dependence. Typically, 100 mL of UO_2_^2+^ solution with an initial concentration of 50 mg·L^−1^ was transferred into 150 mL conical flasks, and the pH was adjusted to 0, 1.0, 2.0, 3.0, 4.0, 5.0, 6.0, and 7.0, respectively. Then, 10 mg of COF-BCTB-Py catalyst was added into each flask, together with 5 mL of methanol as the hole scavenger. The flasks were sealed with sealing film to avoid solvent evaporation. Photocatalytic reactions were conducted at 25°C with magnetic stirring at 300 rpm, using a 300 W xenon lamp equipped with a 420 nm cut-off filter (λ ≥ 420 nm) as the visible light source. After 24 h of irradiation, the suspension was filtered through a 0.22 μm nylon membrane. The concentration of UO_2_^2+^ before and after the reaction was determined by inductively coupled plasma optical emission spectrometry (ICP-OES).

Photocatalytic kinetic experiments were performed to investigate the extraction rate of UO_2_^2+^ over COF-BCTB-Py. Typically, 100 mL of UO_2_^2+^ solution (initial concentration: 180.2 mg·L^−1^, pH = 4) had 10 mg of COF-BCTB-Py and 5 mL of methanol as the hole scavenger added to it. The flask was sealed with sealing film to prevent solvent evaporation. The reaction was carried out under consistent illumination and stirring conditions. At predetermined time intervals (0, 10, 20, 30, 60, 90, 120, 180, 210, 240, 320, 420, 500, 540, 600, and 1440 min), aliquots were withdrawn using a syringe, filtered through a 0.22 μm nylon membrane, diluted appropriately with 3% nitric acid, and analyzed by ICP-OES to determine the residual UO_2_^2+^ concentration.

Adsorption isotherm experiments were conducted to evaluate the maximum uptake capacity. UO_2_^2+^ solutions with initial concentrations of 22.3, 44.1, 86.5, 132.5, 169.4, and 220.5 mg·L^−1^ were prepared and adjusted to pH = 4. In total, 5 mg of COF-BCTB-Py and 5 mL of methanol were added to 100 mL of UO_2_^2+^ solution, followed by sealing to avoid solvent loss. For comparative experiments, 5 mg of 50 kGy-irradiated COF-BCTB-Py and 5 mL of methanol were added to 100 mL of UO_2_^2+^ solution (pH = 4, 220.5 mg·L^−1^). After 24 h of the photocatalytic reaction under identical conditions, the solution was sampled, filtered, diluted with 3% nitric acid, and measured by ICP-OES to quantify the remaining UO_2_^2+^ concentration.

Selectivity experiments were carried out to evaluate the specific recognition capability of COF-BCTB-Py toward UO_2_^2+^. Typically, 5 mg of COF-BCTB-Py was added to 50 mL of aqueous solution containing 0.01 mmol·L^−1^ UO_2_^2+^ and 1 mmol·L^−1^ competing metal ions (including Ba^2+^, Ca^2+^, Co^2+^, Th^4+^, Cd^2+^, Zn^2+^, Na^+^, K^+^, Mg^2+^, and Sr^2+^). The mixture was reacted under the same visible-light photocatalytic conditions for 24 h. Afterward, the supernatant was collected, filtered through a 0.22 μm nylon membrane, diluted appropriately with 3% nitric acid, and analyzed by ICP−OES to determine the concentration variation of UO_2_^2+^.

To investigate the role of methanol as a hole scavenger, control experiments were performed under identical conditions but without the addition of methanol. All other procedures, including catalyst dosage, pH, initial uranium concentration, light source, reaction time, filtration, dilution, and ICP-OES measurement, were kept consistent with those used in the isotherm experiments with sacrificial agents.

To evaluate the practical application potential, photocatalytic removal of UO_2_^2+^ by COF-BCTB-Py was further investigated in natural water matrices, including groundwater and lake water. Each water sample was spiked with 50 mg·L^−1^ uranium to simulate actual radioactive wastewater. Photocatalytic experiments were carried out under identical conditions (pH = 4, 5% methanol as hole scavenger, visible-light irradiation for 24 h). After the reaction, the solution was filtered and analyzed by ICP-OES.

## 5. Conclusions

In summary, we successfully fabricated a novel COF-BCTB-Py with an unconventional D-D configuration, which differs from the conventional D-A structure. The introduction of twisted ligand units modulates the conjugated skeleton and effectively regulates the electronic structure, thereby promoting efficient photogenerated charge separation under visible-light irradiation. Experimental results demonstrate that COF-BCTB-Py enables highly efficient uranyl extraction under weakly acidic to neutral conditions (pH = 4–7), accompanied by excellent structural stability and anti-interference capability. Notably, COF-BCTB-Py exhibits a prominent photocatalytic uranium extraction performance with fast extraction kinetics. Impressively, an ultrahigh uranium removal capacity of 4204 mg·g^−1^ is achieved in high-concentration uranium-containing wastewater, which breaks the conventional cognition that D-D-structured COFs possess inferior photocatalytic capability. Furthermore, the large conjugated skeleton of COF-BCTB-Py endows the material with outstanding structural robustness and highly selective recognition for UO_2_^2+^. The identified uranium product (UO_2_)O_2_·2H_2_O confirms that the uranium immobilization process is dominated by the in situ generation and consumption of hydrogen peroxide. This study proposes an innovative structural design strategy for COF-based photocatalysts and provides new insights into the efficient photocatalytic extraction of uranyl ions from uranium-containing wastewater.

## Data Availability

The original contributions presented in this study are included in the article/Supplementary Materials. Further inquiries can be directed to the corresponding authors.

## Supplementary Materials

The following supporting information can be downloaded at: https://www.mdpi.com/article/10.3390/molecules31132263/s1, including materials and reagents, characterizations, Figure S1: FT−IR data of raw monomers and final products of COF-BCTB-Py. Figure S2: Comparison of photocatalytic uranium extraction performance of COF-BCTB-Py before and after ionizing radiation. Figure S3: PXRD patterns of the photocatalytic product over COF-BCTB-Py without methanol addition. Figure S4: EPR spectra of COF-BCTB-Py. Figure S5: Digital photograph of COF-BCTB-Py.
