# Supplementary material for: Covalent-Organic Framework with Unconventional D-D Structure for Efficient Photocatalytic Uranium Extraction
Source: Molecules. 2026 Jun 26;31(13):2263. doi: 10.3390/molecules31132263 (PMC13362975; doi:10.3390/molecules31132263)
Supplement: Supplementary file 1 [file molecules-31-02263-s001.zip › molecules-4362948-supplementary.pdf]

Supplementary Information (SI)

Covalent–organic framework with unconventional D–D structure for efficient photocatalytic uranium extraction

Dongyang Xu[1,2 †], Xin Du[2 †], Bingyue Zhou[2], Lixi Chen[2,3], Mengyao Li[2], Qiang Wu[3,], Jun Liu[1,*], Songbai Tang[2,*] and Guowen Peng [1,*]*

1 *School of Resources Environment and Safety Engineering, University of South China, 28 Changsheng West Road, Hengyang, 421001, PR China.; xdy17395861820@163.com.*

2 *State Key Laboratory of Radiation Medicine and Protection, School of Radiation Medicine and Protection, Collaborative Innovation Center of Radiological Medicine of Jiangsu Higher Education Institutions, Soochow University, Suzhou, 215123, China.; 20254020010@stu.suda.edu.cn.; lxchen@suda.edu.cn.; 2530509112@stu.suda.edu.cn.*

3 *Key Laboratory of Advanced Nuclear Energy Design and Safety, Ministry of Education, University of South China, 28 Changsheng West Road, Hengyang, 421001, China.*

* *Correspondence: (Q. W) wuqiang@usc.edu.cn.; (J. L) nhliujun@usc.edu.cn.; (S. T) songbtang@suda.edu.cn.; (G. P) pgwnh78@163.com.*

† *These authors contributed equally to this work.*

Materials and reagents

4,4',4'',4'''–(pyrene–1,3,6,8–tetrayl) tetraaniline (Py－NH2, M = 566.69) and 4,4',4'',4'''－([9,9'－dicyclohexyl]－3,3',6,6'－tetrayl)tetrabenzaldehyde (BCTB－CHO, M = 7748.75) were purchased from Jilin Chinese Academy of Sciences Yanshen Technology Co., Ltd. Methanol, 1,4–dioxane, butanol, and o–dichlorobenzene were purchased from Aladdin Industrial Corporation (Shanghai, China). Furthermore, 36% acetic acid was purchased from Energy Chemical Technology (Shanghai) Co., Ltd. All reagents were stored and used according to the instructions and without further purification.

Characterizations

The modeling of COF－BCTB－Py crystal structure, including the stacking theoretical model, powder diffraction data simulation and Pawley refinement, was carried out using Materials Studio version 20.1. The geometric optimization of the COF unit was processed by calculating the minimum energy principle in the software to get the optimal structure during the model building. The structural refinement was conducted by importing the original experimental data into the Forcite module of Materials Studio 20.1 for further refinement. Powder X–ray diffraction (PXRD) data were collected using a Bruker D8 Advance diffractometer employing Cu Kα radiation (λ = 1.54056 Å) in the scanning range of 3~30°. Powder diffraction simulation was performed using Material Studio software. Nitrogen adsorption and desorption isotherms were measured with Quantachrome Autosorb IQ, the specific surface area was calculated from the adsorption data using the Brunauer−Emmett−Teller (BET) method, and the pore size distribution was calculated using the NLDFT method. Calculations were performed with a standard cylindrical pore model for zeolites and silicon－based materials, based on N_2 adsorption data collected at 77 K. Fourier–transform infrared (FT−IR) spectra were collected with a Thermo Nicolet iS50 spectrometer. ^{13}C Solid−state nuclear magnetic resonance (^{13}C SS–NMR) spectra were recorded on a Bruker AVANCE NEO 400 WB (Bruker BioSpin AG, Fällanden, Switzerland). Thermo gravimetric Analysis (TGA) was conducted with a NETZSCH STA 449F3 instrument over the temperature range of 30−900 °C under a nitrogen

atmosphere.SEM images and data were collected using a FEI Quanta 200FEG. The concentrations of UO_2^{2+} solutions were determined by Inductively Coupled Plasma Optical Emission Spectrometry (ICP–OES) (Thermo ICAP 7400). X–ray photoelectron spectroscopy (XPS) was collected on Thermo ESCALAB 250XI America Thermo.

Figures S1–S5

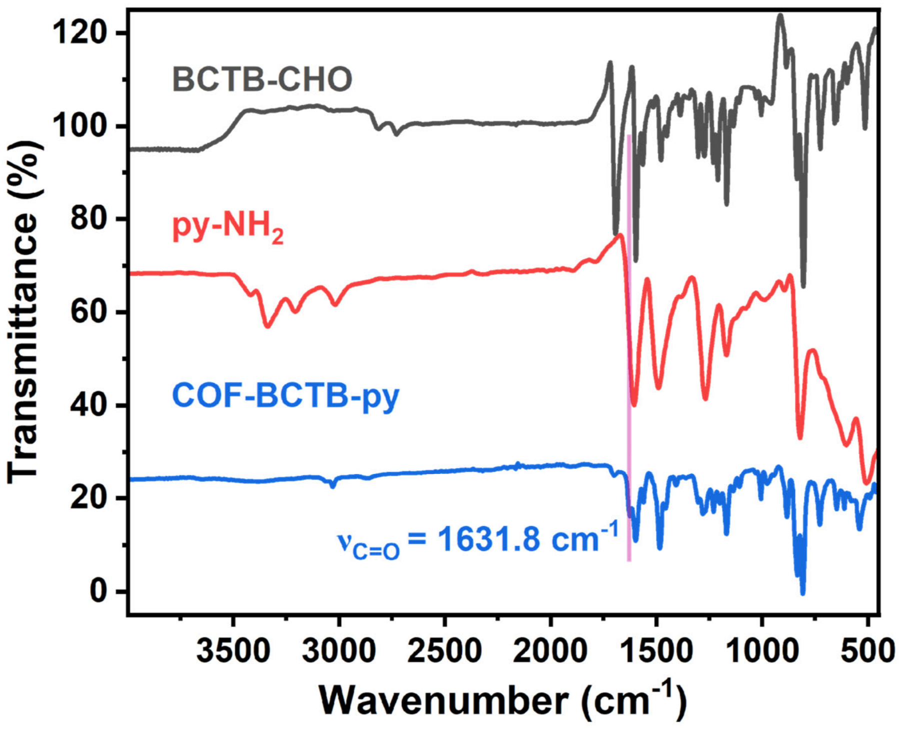

Figure S1. FT−IR data of raw monomers and final products of COF−BCTB−Py.

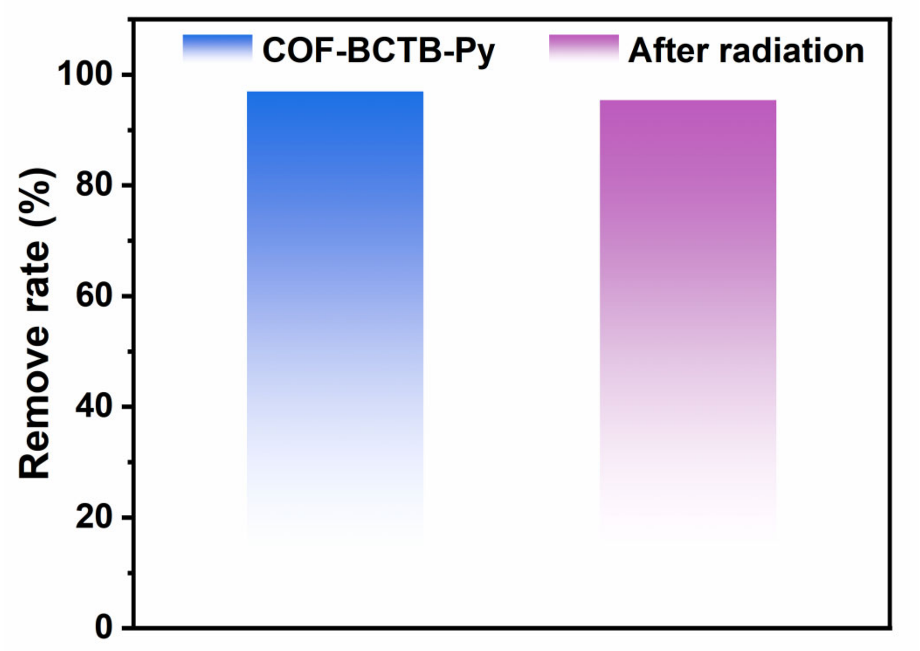

Figure S2. Comparison of photocatalytic uranium extraction performance of COF−BCTB−Py before and after ionizing radiation.

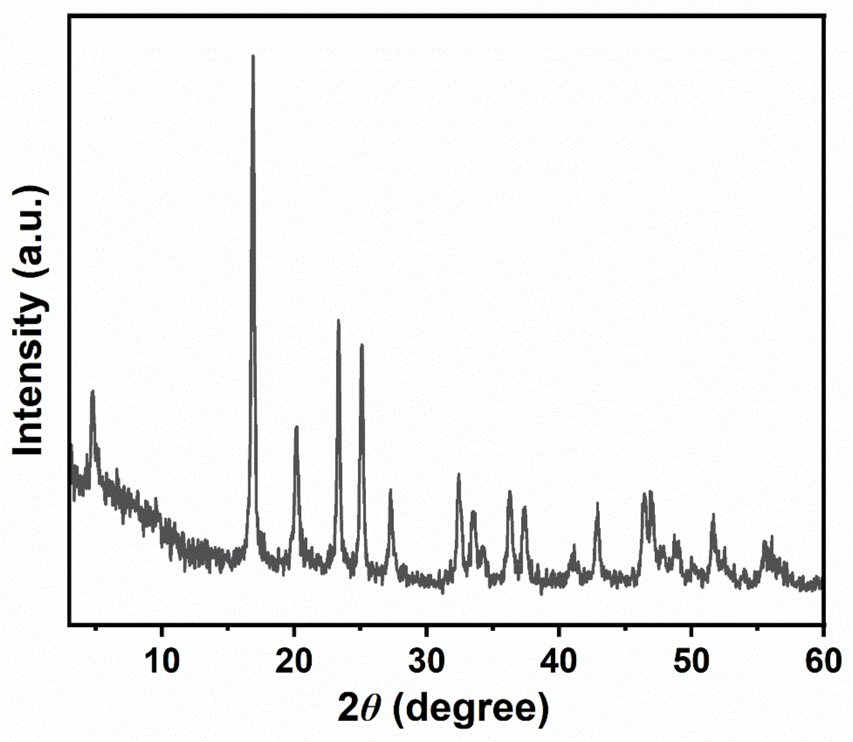

Figure S3. PXRD patterns of the photocatalytic product over COF-BCTB-Py without methanol addition.

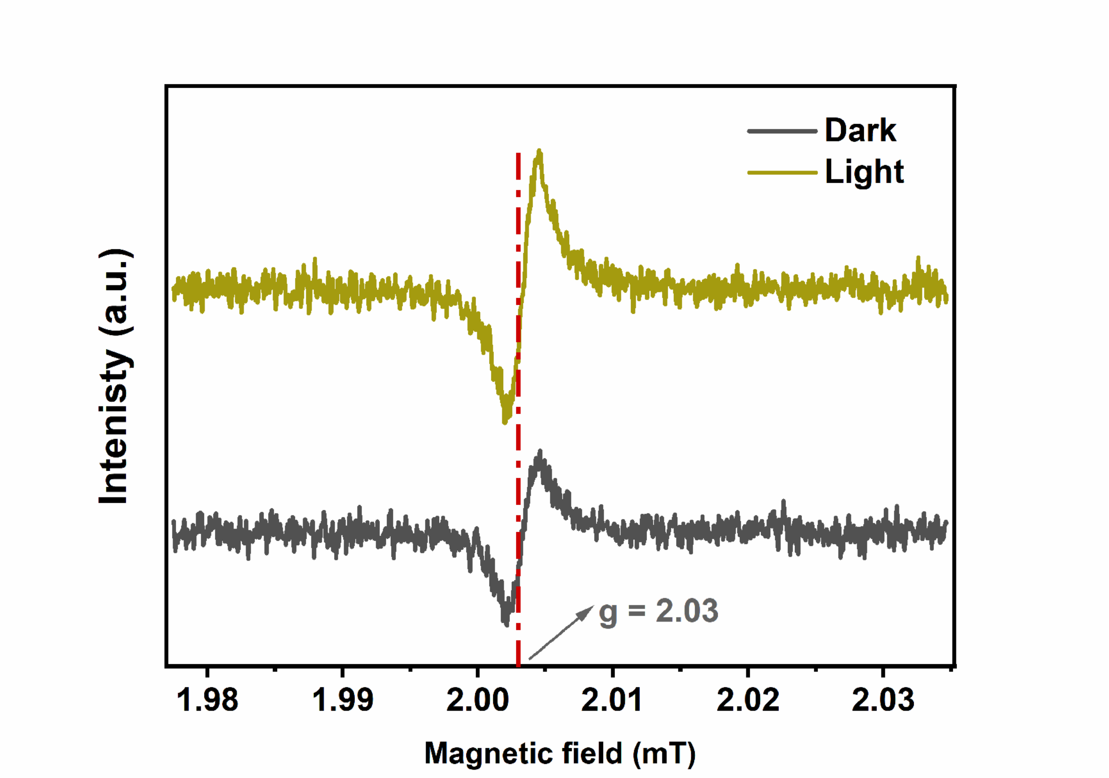

Figure S4. EPR spectra of COF-BCTB-Py.

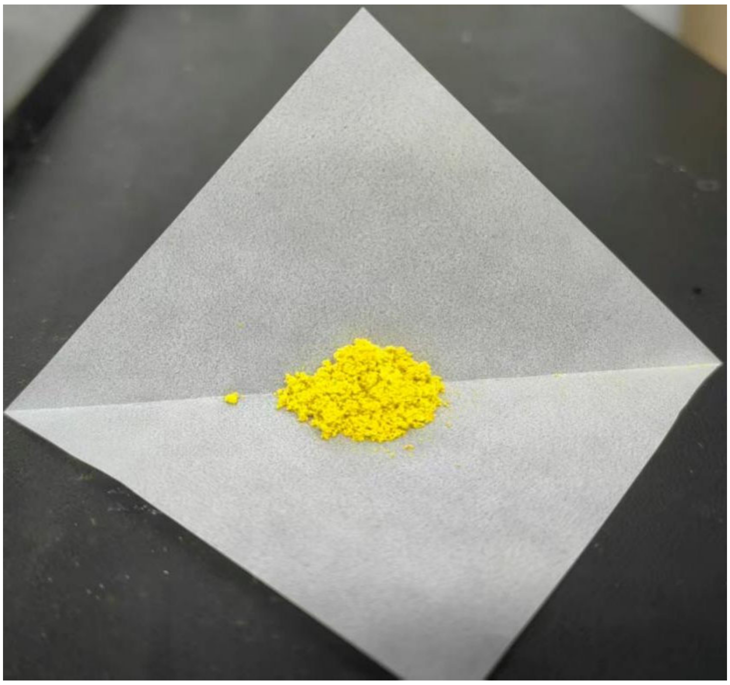

Figure S5. Digital photograph of COF-BCTB-Py.
